# Two case reports of B-cell lymphopenia associated with *IGLL1* variants identified through newborn screening in Ukraine

**DOI:** 10.3389/fped.2025.1566867

**Published:** 2025-05-15

**Authors:** Oksana Boyarchuk, Yaryna Romanyshyn, Ihor Savchak, Volodymyr Kravets, Ivanna Shymanska, Halyna Makukh

**Affiliations:** ^1^ Department of Children’s Diseases and Pediatric Surgery, I. Horbachevsky Ternopil National Medical University, Ternopil, Ukraine; ^2^ Clinic of Pediatric Immunology and Rheumatology, Western Ukrainian Specialized Children’s Medical Centre, Lviv, Ukraine; ^3^ Department of the Research and Biotechnology, Scientific Medical Genetic Center LeoGENE, Lviv, Ukraine; ^4^ Department of Genetics and Biotechnology, Ivan Franko National University of Lviv, Lviv, Ukraine

**Keywords:** B-cell lymphopenia, *IGLL1* gene, newborn screening, KREC, diagnosis, autosomal-recessive agammaglobulinemia, inborn errors of immunity

## Abstract

Before the implementation of newborn screening (NBS), only a few cases of agammaglobulinemia associated with *IGLL1* variants had been reported. The *IGLL1* gene encodes the surrogate light chain components *λ*5 and VpreB, which form a crucial part of the pre-B cell receptor complex. A recently published study reported 17 cases of agammaglobulinemia caused by *IGLL1* variants, the vast majority of which were identified through NBS. Here, we report two cases of B-cell lymphopenia along with *IGLL1* variants identified through NBS in Ukraine. Both neonates had undetectable KREC and normal TREC levels at birth. Despite the presence of B-cell lymphopenia, only one patient exhibited a transient decline in IgG levels. IgA and IgM levels remained normal during the first year of follow-up, which had not been reported in previous *IGLL1* cases. Both children presented with mild upper respiratory tract infections. Genetic analysis revealed that both patients carried the c.425C > T variant, with one patient also harboring the c.258del variant. These variants have been linked to B-cell lymphopenia and low KREC levels in prior studies. Two additional variants were identified on the second chromosome: c.368C > G, which is predicted to be tolerated, and c.377T > C, which is likely disruptive. This study highlights the potential underdiagnosis of B-cell lymphopenia caused by *IGLL1* variants. Moreover, the comparison between clinically diagnosed cases and those identified through NBS underscores the importance of early diagnosis that facilitates close monitoring of affected patients from birth, timely initiation of immunoglobulin replacement therapy, and the prevention of complications and severe manifestations.

## Introduction

Predominantly antibody deficiencies encompass a group of inborn errors of immunity (IEI) characterized by profoundly decreased or absent B cells (1). These deficiencies include X-linked agammaglobulinemia (XLA), also known as Bruton's tyrosine kinase (BTK) deficiency; certain types of autosomal-dominant immunodeficiencies (such as Hoffman syndrome, PU1 deficiency, and E47 transcription factor deficiency), and several autosomal-recessive immunodeficiencies. Among the autosomal-recessive diseases are deficiencies in components critical to B-cell development, including µ heavy chain, lambda-5 (λ5), Igα, Igβ, BLNK, p110δ, p85, E47 transcription factor, SLC39A7, and FNIP1 deficiencies.

XLA is the most common congenital agammaglobulinemia, accounting for approximately 85% of cases (2). Other variants of agammaglobulinemia are considerably rarer. It has been suggested that autosomal-recessive agammaglobulinemias (ARA) generally have a more severe clinical course and manifest at an earlier age compared to XLA (3).

Before the implementation of newborn screening (NBS), only a few cases of agammaglobulinemia associated with *IGLL1* variants had been reported (2, 4, 5). The *IGLL1* gene encodes the surrogate light chain λ5 and VpreB, forming a crucial pre-B cell receptor complex (pre-BCR) component. The pre-BCR complex initiates key processes in pre-B cells that ultimately lead to their differentiation into immature B cells (6). Variants in components of the pre-B cell receptor and BCR complex account for approximately 5%–7% of patients with defects in early B-cell development (3).

Using kappa-deleting recombination excision circles (KREC) assays in newborn screening programs has facilitated the identification of conditions associated with B-cell lymphopenia (7, 8, 9, 10). However, including KREC assays in routine NBS programs remains controversial and less widespread than the TREC assay, which is used globally to detect severe combined immunodeficiency (SCID).

A recently published study reported 17 cases of agammaglobulinemia caused by *IGLL1* variants, 13 of which were diagnosed through NBS (11). This study significantly expanded the known clinical phenotypes associated with *IGLL1*-related agammaglobulinemia.

The aim of our study was to present two additional cases of B-cell lymphopenia associated with *IGLL1* variants identified through NBS in Ukraine to highlight the importance of early detection and further support the consideration of the KREC assay for global implementation in newborn screening programs to identify early B-cell development defects.

## Materials and methods

### Clinical evaluation

The patients were monitored by immunologists following positive newborn screening (NBS) results. The study adhered to the principles of the 1975 Declaration of Helsinki (as amended in 2000) and received approval from the Ethics Committee of I. Horbachevsky Ternopil National Medical University. Informed consent was obtained from the legal guardians of all participants.

### Blood cells and immunological studies

Routine hematological assays were performed for complete blood cell analysis. Peripheral blood mononuclear cell lymphocyte subsets were identified via flow cytometry. Monoclonal antibodies were used to detect cell surface markers, including CD3, CD4, CD8, CD19, CD16, and CD56. Serum levels of IgG, IgA, IgM, and antibodies to diphtheria and tetanus toxins were measured using standard immunological techniques.

### Whole exome sequencing (WES) and panel sequencing

Whole exome sequencing (WES) for the second case was conducted at the Scientific Medical Genetic Center LeoGENE, Lviv, Ukraine. For the first case, panel sequencing targeting inborn errors of immunity and cytopenias was performed at Invitae Laboratory, focusing on 574 primary immunodeficiency-related genes. Genomic DNA was enriched for targeted regions using a hybridization-based protocol and sequenced with Illumina technology at a coverage depth of ≥50x. Reads were aligned to a reference genome, and clinically significant findings were confirmed using orthogonal validation methods.

### Identification of published *IGLL1* cases

We conducted a scoping search to identify additional reported cases of IGLL1 deficiency. The search was performed using PubMed and Scopus databases. We used the following search terms: “*IGLL1*,” “*IGLL1* deficiency,” “*IGLL1* mutation,” and “*IGLL1* variant.” The search included articles published up to January 2024. Reference lists of relevant articles were also manually screened to identify additional cases. Only peer-reviewed case reports and original articles describing patients with pathogenic or likely pathogenic *IGLL1* variants were included.

## Results

### Case presentation

**Case 1:** A full-term, healthy female newborn had a positive NBS result with undetectable KREC in the first and second (retest) dried blood spot (DBS) samples but normal TREC levels [Ct = 33.2 (164 copies in DBS) and Ct = 28.4 (3,850 copies in DBS), respectively] at birth. Birth weight was 4,500 g. Follow-up revealed low B cell counts (70 cells/μl) but preserved T and NK cell subsets at 3 months (Table 1). Immunoglobulin levels (IgA, IgM, and IgG) remained within normal ranges at 3, 6, 10, and 15 months of age. Slightly reduced CD8 and low CD19 levels were observed at 6 months. By 10 months, CD3, CD4, and CD8 levels were within normal ranges, and an increase in CD19–128 cells/µl was noted. B-cell phenotyping was conducted at the age of 1 year and 3 months. Naïve and memory B-cells were within normal range (Table 1). No severe infections occurred until 1 year 4 months of age.

**Table 1:** Immunologic parameters in the presented cases.

Parameter	Case 1				Case 2			Normal range
Age, months	3	6	10	15	1	3.5	7	
CD3, %	85.7	88.8	83.1	86	71.57		86.1	50–76
CD3, cells/µl	3,330	2,620	2,720	3,720	4,798		4,476	1,800–6,500
CD4, %	63.8	72.2	61.6	59.8	45.7		58.1	35–57
CD4, cells/µl	2,570	2,010	2,090	2,490	3,065		3,021	1,200–4,600
CD8, %	19.1	**14** **.** **6**	21.5	23.7	23		24.6	16–34
CD8, cells/µl	770	**410**	730	990	1,542		1,279	700–2,400
CD19, %	**2**	**1** **.** **8**	**4** **.** **1**	**6** **.** **14**	**1** **.** **4**		**1** **.** **3**	17–32
CD19, cells/µl	**70**	**57**	**128**	**176**	**97**		**68**	500–2,200
B1-cells, % (CD45 + CD19 + CD5+)				**3** **.** **5**				4–17
B2-cells (naïve), % (CD45 + CD19 + CD5-CD27-)				84.3				80–96
B-cells, memory, % (CD45 + CD19 + CD5-CD27+)				12.1				10–40
CD16/56, %	11.6	8.3	10.3	7.9	25		12,6	4–16
CD16/56/mcl	440	258	325	352	1,720		655	100–900
IgA, g/L	0.15	0.17	0.25	0.30	0.06	0.07	0.84	0.02–0.83
IgM, g/L	0.64	0.64	1.27	0.97	0.03	0.46	0.41	0.03–1.45
IgG, g/L	3.9	2.5	4.2	7.2	4.3	**1** **.** **77**	2.5	2.32–14.11
IgE, IU/ml	<1.5	3.0	3.3				5.4	<8
Complement activity, CH50	67	65	80	57				19–65
Antibodies to diphtheria toxoid IgG, U/ml		**0** **.** **029**	**0** **.** **020**	**0** **.** **014**				<0.01 negative
								0.01–0.099 doubtful
Antibodies to tetanus toxoid IgG, U/ml		0.28						<0.10 negative
								>0.11 positive

Indicators that do not fall within the normal range are highlighted in bold.

Vaccine responses were notable for normal tetanus antibody titers but borderline diphtheria titers. Genetic testing identified variants of uncertain significance (VUS) in the *IGLL1* gene: one allele with c.425C > T (p.Pro142Leu) and the other with c.368C > G (p.Ser123Cys) and c.377T > C (p.Leu126Pro).

**Figure 1:**
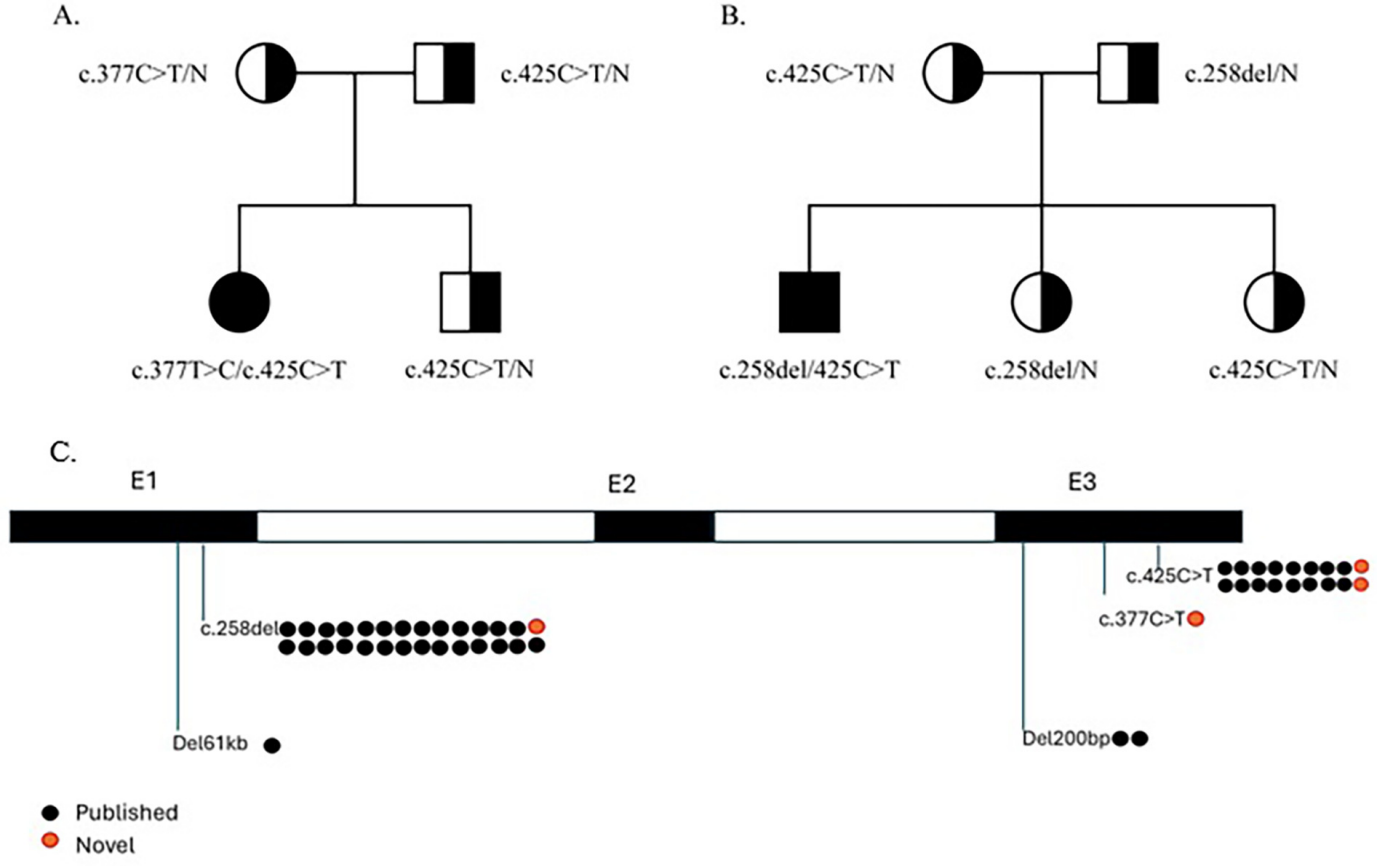
Family pedigree of the presented cases and *IGLL1* variants. **(A)** Family pedigree of case 1. **(B)** Family pedigree of case 2. **(C)** *IGLL1* variants that were detected in previously described patients and presented patients.

Family genetic testing revealed that the parents and one sibling were carriers (Figure 1A).

**Case 2:** A full-term, healthy male newborn had a positive NBS result with undetectable KREC in the first DBS sample and low KREC level [Ct = 39.2 (16 copies in DBS)] in the second DBS (retest) but normal TREC levels [Ct = 32.6 (159 copies in DBS) and Ct = 33.2 (164 copies in DBS), respectively] at birth. Birth weight was 3,450 g. Follow-up revealed low B cell counts (97 cells/μl) but preserved T and NK cell subsets at one month (Table 1). B-cell counts decreased to 68 cells/μl at seven months, while T-cell counts remained normal. A transient IgG decline was noted at 3.5 months, with levels increasing to low-normal by 7 months. The patient did not receive vaccination due to parental refusal.

In a complete blood count (CBC) at 7 months, anemia (Hb—108 g/L) and thrombocytosis (758 G/L) were observed, while neutrophil and lymphocyte levels were normal. Genetic testing revealed two *IGLL1* variants on separate alleles: a missense VUS c.425C > T (p.Pro142Leu) and a likely pathogenic nonsense variant c.258del (p.Gln88Asnfs*7). Family genetic testing revealed that parents and two siblings are carriers (Figure 1B). Immunoglobulin replacement therapy (IgRT) was recommended and initiated at the age of 1 year and 3 months. Over one year, the child experienced mild respiratory symptoms and transient fever episodes.

At the time of the last clinical follow-up, the first patient was 1 year and 4 months old, and the second patient was 1 year and 5 months old. Both patients remain under regular supervision by a clinical immunologist and a pediatrician.

## Discussion

In the cases we presented, B-cell lymphopenia, along with *IGLL1* variants, was identified through NBS. Following a pilot project conducted in 2020–2022 (9, 12), population-based NBS for SCID and severe B-cell lymphopenias was officially launched in Ukraine in October 2022 (13). The program initially operated at two centers (Lviv and Kyiv), covering 12 out of the country's 25 regions. In April 2023, two additional centers (Kharkiv and Kryvyi Rih) joined the initiative, expanding coverage to more regions, except for territories occupied by Russia.

During this period, more than 300,000 newborns were screened. The KREC assay enabled the early diagnosis of four newborns with XLA and two with ARA associated with *IGLL1* variants. Additionally, three cases of Nijmegen breakage syndrome were identified, characterized by low TREC and KREC levels (14).

Both cases of B-cell lymphopenia associated with *IGLL1* variants were detected in the western regions of Ukraine (Lviv and Ternopil). Based on cases identified by NBS, the incidence of B-cell deficiency due to *IGLL1* variants in Austria, Czechia, and Switzerland was estimated to be at least 1.3 per 100,000 births (11). In Ukraine, the frequency is lower, approximately 1 in 150,000 newborns. However, considering that both cases were identified in western regions, the incidence in this area aligns with the previously reported rate of 1–2 per 100,000 births.

Both patients carried the c.425C > T variant, and one also had the c.258del variant, which was recently reported with relatively high allele frequencies in the general population (0.09% and 0.13%, respectively). However, these variants have also been linked to several cases of B-cell lymphopenia and low KREC levels (11). Other reported cases similarly identified these variants in either homozygous or heterozygous states (4, 5).

Twelve out of 13 reported ARA cases caused by *IGLL1* variants identified by NBS were associated with these specific variants: two homozygous for c.425C > T, two homozygous for c.258del, and the rest compound heterozygous (11). According to ACMG criteria, variant c.425C > T can be classified as likely pathogenic, because its frequency is extremely low in the general population (0,09%), which is allowed for a recessive disorder that does not cause a selective effect in carriers [criterion PM2 (Moderate)]; detected in trans with a pathogenic variant (11) [criterion PM3 (Moderate)], multiple lines of computational evidence support a deleterious effect on the gene product (supporting criterion PP3), and patients phenotype is highly specific for a disease with a single genetic etiology (supporting criterion PP4). In conclusion, this variant has 2 moderate and 2 supporting criteria, and we can classify this variant as likely pathogenic (15). All *IGLL1* variants that were detected in previously described patients and presented patients are shown in Figure 1C.

In the second case we described, two additional variants were found on the second chromosome: c.368C > G (p.Ser123Cys) and c.377T > C (p.Leu126Pro). The frequency of c.377T > C (p.Leu126Pro) in the general population is 0.06%. PolyPhen-2, a predictive algorithm assessing the impact of missense mutations on protein structure and function, suggests that this variant is likely disruptive, whereas the c.368C > G (p.Ser123Cys) variant is likely to be tolerated. The latter is present in population databases (rs537809626, gnomAD 0.01%).

A comparison of baseline and clinical characteristics of children with ARA associated with *IGLL1* variants, identified through NBS (11) and the newborns reported here (Table 2), demonstrated a similar mild clinical course. In both cases, only mild upper respiratory tract infections (URTI) were observed.

**Table 2:** Comparison of cases' and previously described patients' characteristics with *IGLL1* variants identified by NBS.

Characteristic	Described patients	Case 1	Case 2
	*n* = 13 (11)		
Baseline characteristic	Me (range) or *n* (%)		
Age at immunologic diagnosis, weeks	3 (2–18)	11	4
Preterm birth	1 (7.7)	No	No
Birth weight, g	3,225 (2,070–4,100)	4,500	3,450
Sex	M/F—7/6	F	M
Clinical features			
Mild URTI	10 (76.9)	Yes	Yes
Mild GI infection	2 (15.4)	No	No
Atopy	5 (38.5)	No	No
Autoimmunity	No	No	No
Malignancy	1 (7.7)	No	No
Syndromic features	2 (15.4)	No	No
Transient neutropenia	11 (84.6)	NA	No
Thrombocytosis at first year of life	13 (100)	No	Yes
B-cells range during first year of life, cells/µl	0–230	57–128	68–97
Normal IgG level at initial investigation	12 (92.3)	Yes	Yes
		3.9 g/L	4.3 g/L
Low IgM level at initial investigation	12 (92.3)	No (0.64 g/L)	0.03 g/L (lower limit of normal)
Consistently low IgA level	13 (100)	No	No
		(0.15–0.25) g/L	(0.06–0.84) g/L
Genetic diagnosis			
Age at genetic diagnosis, months	2 (1–6)	9	4
Methods	WES—7 (53.8), Gene panel—6 (46.2)	Gene panel	WES
Zygosity	Homozygous—5 (38.5)	Compound heterozygous	Compound heterozygous
	Compound heterozygous—7 (53.8)		
	Unknown—1 (7.7)		
Treatment			
IgRT	12 (92.3)	No	Yes
Age at IgRT, months	4.5 (1–6)	-	15
Previously reported	1 (7.7)	No	No

M, male; F, female; URTI, upper respiratory tract infection; GI, gastroenterological; IgRT, immunoglobulin replacement therapy; WES, whole exome sequencing.

We did not notice transient neutropenia, which was relatively common in the previously reported cohort (84.6%). Thrombocytosis was detected in only one child. Lymphocyte subset counts, as well as IgG levels during the initial investigation, were comparable to previously published findings (11), including B-cell counts. However, in our patients, IgA levels were within age-appropriate norms, while IgM levels were at the lower limit of normal in one patient. To date, our patients are 1 year and 4 months old (case 1) and 1 year and 5 months old (case 2). Only one of them (case 2) has initiated IgRT, which was started at the age of 1 year and 3 months.

Overall, patients with B-lymphopenia associated with *IGLL1* variants can be divided into three cohorts based on the diagnostic method (11): patients identified through NBS using the KREC assay, clinically diagnosed patients, and those identified as siblings or parents (Table 3).

**Table 3:** Comparison of cases with IGLL1 variants identified by NBS, clinically diagnosed, and diagnosed in siblings or parents.

Characteristic	Patients identified by NBS	Clinically diagnosed	Diagnosed in siblings or parents
	*n* = 15	*n* = 6	*n* = 4
Baseline characteristic	Me (range) or *n* (%)		
Age at immunologic diagnosis	3 (2–18) weeks	2.5 (0.2–8) years	8.5 (2–34) years
Sex, M/F	8/7	3/3	1/3
Clinical presentation			
Symptomatic	14 (93.3)	6 (100)	1 (25.0)
Mild URTI	12 (80.0)	3 (50.0)	No
LRTI	0	4 (66.7)	No
Mild GI infection	2 (13.3)	1 (16.7)	No
Other infections	No	2 (33.4)	1 (25.0)
		Meningitis, prolonged varicella, UTI, sepsis	Conjunctivitis
Complications	No	2 (33.4)	No
		Bronchiectasis, conductive hearing loss, peritonitis	
Atopy	5 (33.3)	No	1 (25.0)
Autoimmunity	No	No	No
Malignancy	1 (6.7)	No	No
Syndromic features	2 (13.3)	1 (16.7)	No
	Duplex kidney, ASD	Exocrine pancreatic insufficiency, failure to thrive, unclear degenerative muscle disease and neuropathy	
Transient neutropenia	11 (73.3)	1/2 (50.0)	No
Thrombocytosis at first year of life	14 (93.3)	1/2 (50.0)	NA
B-cells range, cells/µl	0–230	0–94	18–167
Normal IgG level at initial investigation	14 (93.3)	1 (16.6)	2/2 (100)
Low IgM level at initial investigation	13 (86.7)	6 (100)	0/2 (0)
Low IgA level	13 (86.7)	6 (100)	0/2 (0)
Genetic diagnosis			
Age at genetic diagnosis	2 (1–6) mos	3.5 (0,6–15) years	9.5 (2–34) years
Zygosity	Homozygous—5 (33.3)	Homozygous (c.258delG)	Homozygous (c.258delG)
	Compound heterozygous—9 (60.0)	–3 (50.0)	–3 (75.0)
		Compound heterozygous—3 (50.0)	Compound heterozygous—1 (25/0)
	Unknown—1 (6.7)		
Treatment			
IgRT	13 (86.7)	6 (100)	0 (0)
Age at IgRT, months	4.5 (1–15)	21.5 (3–96)	No
References	11, presented cases	4, 5, 16, 17, 18	11, 16, 17

NBS, newborn screening; M, male; F, female; URTI, upper respiratory tract infection; LRTI, lower respiratory tract infection; GI, gastrointestinal; UTI, urinary tract infection; ASD, autistic spectrum disorder; IgRT, immunoglobulin replacement therapy; WES, whole exome sequencing.

A comprehensive analysis of all reported literature cases to date identified 25 patients with B-cell lymphopenia and *IGLL1* variants. Only documented cases, including the two described in this study, were considered. The largest cohort consists of 15 patients identified through NBS, followed by 6 clinically diagnosed patients, 3 identified in siblings, and 1 case in a symptomatic mother of affected patients.

A comparison of the three cohorts revealed a broad spectrum of clinical manifestations associated with *IGLL1* variants. The median age at immunological and genetic diagnosis in the clinically diagnosed cohort was 2.5 and 3.5 years, respectively, whereas screening enabled case detection within the first weeks of life. Accordingly, one-third of clinically diagnosed patients experienced severe infections (e.g., meningitis, prolonged varicella, urinary tract infection, sepsis) and complications (e.g., bronchiectasis, conductive hearing loss, peritonitis). In one case, immunodeficiency was suspected only after the onset of bronchiectasis.

Early detection through screening allowed for the initiation of IgRT at a significantly younger age (4.5 months vs. 21.5 months), which could potentially reduce the risk of complications and improve the quality of life in affected children.

Clinically diagnosed cases (4, 5, 16, 17, 18) demonstrated a more pronounced immunological phenotype at presentation. These patients had significantly lower B-cell counts (often undetectable), reduced levels of IgM and IgA in all cases, and decreased IgG levels in 83.4% of patients. In contrast, patients diagnosed via screening showed greater variability in B-cell counts and immunoglobulin levels.

Among the four individuals identified through family screening, three were siblings (aged 22, 2, and 9 years) and one was a mother (34 years old). KREC levels were not determined in any of these asymptomatic carriers. At the same time, none of the patients in whom the identified *IGLL1* variant was detected during the examination of siblings or parents (11, 16, 17) exhibited any symptoms except for one case of moderate atopic dermatitis at the age of 14 years. Despite B-cell lymphopenia, immunoglobulin levels remained within normal ranges in all individuals of this cohort. References (11, 16, 17) do not provide data regarding vaccine responses in such individuals.

All groups included both homozygous and compound heterozygous cases. No clear genotype-phenotype correlations were observed.

The presence of asymptomatic individuals carrying homozygous IGLL1 c.258delG variants supports the notion of incomplete penetrance associated with IGLL1 mutations. This phenomenon may be influenced by additional genetic, epigenetic, or environmental modifiers that modulate the clinical expression of the defect. Further studies, including detailed immunophenotyping and longitudinal follow-up, are necessary to better understand the variability in clinical presentation and immune function among carriers.

Thus, the cases presented here expand the data on immunological changes and genetic variants associated with B-cell lymphopenia linked to *IGLL1* variants, identified through NBS. Notably, normal levels of IgA and IgM were observed, which had not been reported in previous similar cases.

The early identification of B-cell lymphopenia through neonatal screening has had a significant impact on the clinical care of the two patients presented in this study. From the parents' viewpoint, receiving a positive NBS result for an immunodeficiency was initially unexpected and distressing, particularly in the absence of clinical symptoms. However, structured follow-up by a multidisciplinary team—including immunologists and pediatricians—provided reassurance and clear guidance throughout the diagnostic and monitoring process.

For case 1, regular assessments and the absence of infections helped reinforce the decision to adopt a watchful waiting approach without initiating immunoglobulin replacement therapy. In contrast, case 2 demonstrated more pronounced immunological changes, leading to the initiation of IgRT at 1 year and 3 months, which the family accepted as a preventive measure to reduce the risk of infections.

Importantly, both families expressed appreciation for the benefits of early detection, emphasizing their ability to make informed decisions regarding treatment, vaccination, and daily care. These cases underscore how NBS enables personalized management and supports caregiver involvement from the earliest stages of life.

The limitation of our study is the short follow-up of the two infants reported. Further monitoring will allow us to identify the impact of new *IGLL1* variants on clinical manifestation and immunological parameters.

Thus, this study highlights the potential underdiagnosis of B-cell lymphopenia secondary to *IGLL1* variants. Furthermore, the comparison between clinically diagnosed cases and those identified through neonatal screening underscores the importance of early diagnosis. Early detection allows for close monitoring of these patients from birth, timely initiation of IgRT, and prevention of complications and severe manifestations.

Identification of ARA associated with *IGLL1* variants through neonatal screening, along with long-term monitoring of affected patients, will expand our understanding of the disease's course and improve care for these patients.

## Data Availability

The original contributions presented in the study are included in the article/supplementary material, further inquiries can be directed to the corresponding author.
